# Pulse Based Time-of-Flight Range Sensing

**DOI:** 10.3390/s18061679

**Published:** 2018-05-23

**Authors:** Hamed Sarbolandi, Markus Plack, Andreas Kolb

**Affiliations:** Institute for Vision and Graphics, University of Siegen, 57068 Siegen, Germany; markus.plack@student.uni-siegen.de (M.P.); andreas.kolb@uni-siegen.de (A.K.)

**Keywords:** time-of-flight, pulse-based, range sensor, computer vision

## Abstract

Pulse-based Time-of-Flight (PB-ToF) cameras are an attractive alternative range imaging approach, compared to the widely commercialized Amplitude Modulated Continuous-Wave Time-of-Flight (AMCW-ToF) approach. This paper presents an in-depth evaluation of a PB-ToF camera prototype based on the Hamamatsu area sensor S11963-01CR. We evaluate different ToF-related effects, i.e., temperature drift, systematic error, depth inhomogeneity, multi-path effects, and motion artefacts. Furthermore, we evaluate the systematic error of the system in more detail, and introduce novel concepts to improve the quality of range measurements by modifying the mode of operation of the PB-ToF camera. Finally, we describe the means of measuring the gate response of the PB-ToF sensor and using this information for PB-ToF sensor simulation.

## 1. Introduction

Computer vision research and its application is exponentially growing in terms of using and incorporating new sensor modalities and, thus, it naturally profits from improvements in sensor developments. Depth sensing cameras capable of real-time range acquisition have been investigated for more than 10 years. In recent years, amplitude modulated continuous-wave Time-of-Flight (AMCW-ToF) cameras became very popular, mainly due to the second generation of the Kinect™ based on this technology, i.e., the Kinect${}^{\mathrm{ToF}}$. Besides very popular applications such as Human-Computer-Interaction (HCI) and gaming, other applications such as human body and animal detection [1], physical rehabilitation [2], or surveillance [3] strongly benefit from real-time and robust ToF cameras. Even though AMCW-ToF cameras are very mature already, they comprise a large amount of specific characteristic properties which are intrinsic to the technology itself. Examples are multi-path effects, motions artifacts, and systematic distance errors [4].

The pulse-based Time-of-Flight (PB-ToF) principle is an alternative range sensing approach [5]. Depending on the specific application under consideration, the pulse based concept may be preferable to AMCW-ToF due to its different intrinsic characteristics. Various publications deal with the evaluation of AMCW-ToF cameras [4,6], the comparison between models of different manufacturers [7], and the comparison of AMCW-ToF cameras with other range imaging devices such as stereo systems [8], and structured light cameras (mainly the first Kinect version) [9,10,11,12]. We are not aware of any in depth evaluation of any kind of PB-ToF-camera, which might be due to their rather restricted availability.

In this paper we investigate the intrinsic characteristics of a PB-ToF camera prototype based on the Hamamatsu area sensor S11963-01CR. After discussing the PB-ToF principal we evaluate the following characteristic error sources using the methodology proposed by Sarbolandi et al. [4]:

- Temperature drift,
- Depth inhomogeneity and linearity error,
- Dynamic scenery, and
- Multi path effects.

Beyond this, we introduce a novel concept in evaluating the PB-ToF’s behavior with respect to the non-ideal pulse shapes. We demonstrate how to apply this concept to optimize the cameras point of operation of PB-ToF cameras with the goal to improve the robustness of the range measurement. Furthermore, we use the captured signals and validate the PB-ToF sensor model by comparing simulated and measured results.

## 2. ToF Range Measurement Principle

The range measurement using ToF technology is based on the time difference that emitted light takes to travel to the object and bounce back to the sensor unit [13]. As the speed of light in vacuum $c_{0}=299.792\times 10^{6}\;\frac{m}{s}$ is constant (and nearly the same as in air), the distance *d* is linearly related to the half of the travel time ΔT yielding:

(1) $$d=\frac{1}{2}\;c_{0}\Delta T.$$

This assumes that both sensor and illumination unit have the same location, which is physically impossible. Practically, the sensor and illumination unit are placed as close to each other as possible.

There are different principles to measure ΔT, out of which two will be presented in the following, i.e., the *Amplitude Modulation Continuous Wave (AMCW)* and the *Pulse Based (PB)* approach. In the first method a sinusoidal intensity modulated signal is emitted and the phase shift in the intensity of the reflected light is measured, whereas the second approach emits very short light pulses in combination with a synchronized gate imager. Depending on the delay of the received light pulses, the portion of the photons accumulated by the gate imager is proportional to the distance.

### 2.1. Amplitude Modulated Continues-Wave ToF (AMCW-ToF)

**Figure 1:**
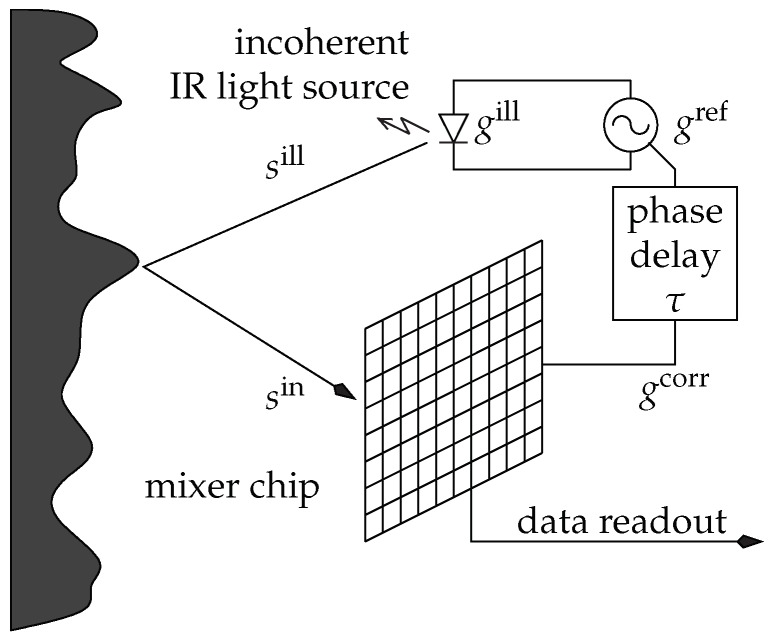
The time of flight (ToF) phase-measurement principle [4].

The AMCW approach is the most commonly used approach for ToF cameras. In the following, we will mainly follow the notation from [4]. The general operation principle is to actively illuminate the scene using periodically intensity modulated near infrared (NIR) light (see Figure 1). The distance between sensor and the observing scene point induces a *time shift* in the reflected optical signal which is equivalent to a *phase shift* ϕ[rad] in the periodic signal, which is assumed to have a *modulation frequency* $f_{m}$. The phase shift is detected in each sensor pixel by a so-called *mixing* process. This time shift can be easily transformed into the sensor-object distance as the light has to travel the distance twice, i.e., $d=\frac{c_{0}\phi }{4\pi f_{m}}$.

From the technical perspective, the generator signal $g^{\mathrm{ref}}$ driving the illumination unit results in the intensity modulated signal $g^{\mathrm{ill}}$ which, after being reflected by the scene, results in an incident optical signal $s^{\mathrm{in}}$ on each sensor pixel. Note that the optical signal may be deformed by nonlinear electronical effects e.g., in the LEDs of the illumination unit. The phase shift is computed using several correlation measurements with varying additional phase offset τ and, optionally, with different frequencies. The measurements at specific phase shifts are frequently called *phase image* or *correlation image*. Practically, the correlation images are acquired sequentially, however, there is the theoretic option to acquire several correlation images in parallel. Note that due to the periodicity of the reference signal, any ToF-camera has a unique unambiguous measurement range.

Commonly, AMCW-ToF cameras assume a sinusoidal reference signal $g^{\mathrm{ref}}\left(t\right)=\cos\left(2\pi f_{m}t\right)$ with a single modulation frequency $f_{m}$ and an illumination signal $g^{\mathrm{ill}}$ proportional to $g^{\mathrm{ref}}$. For a given internal phase offset $\tau_{i}$ the *correlation image* then results from the convolution-like integration of the incident light signal $s^{\mathrm{in}}$ with the *correlation signal* $g^{\mathrm{corr}}$, which is the reference signal shifted by the phase offset $\tau_{i}$, i.e., $g^{\mathrm{corr}}\left(t\right)=g^{\mathrm{ref}}\left(t+\tau \right)$, yielding

$$\begin{array}{cc} A & =\lim_{T\to \infty }\int_{-T/2}^{T/2}s^{\mathrm{in}}\left(t\right)g^{\mathrm{corr}}\left(t\right)\;dt\\ & =\lim_{T\to \infty }\int_{-T/2}^{T/2}s^{\mathrm{in}}\left(t\right)g^{\mathrm{ref}}\left(t+\tau \right)\;dt. \end{array}$$

Commonly, four correlation images $A_{i}$ with $\tau_{i}=\frac{\pi }{2}i,\;i=0,\ldots ,3,$ are acquired leading to the following computation of the distance related phase shift

(2) $$\phi =\mathrm{error}(A_{3}-A_{1},A_{0}-A_{2}).$$

Here, arctan 2(y,x) is the angle between the positive *x*-axis and the point given by the coordinates (x,y).

The Kinect${}^{\mathrm{ToF}}$ camera also applies the AMCW intensity modulation approach [14]. Blake et al. [15] reverse engineered the Kinect${}^{\mathrm{ToF}}$-driver. This revealed that the Kinect${}^{\mathrm{ToF}}$ acquires 10 correlation images, from which nine correlation images are used for a three-phase reconstruction approach based on phase shifts of $0^{\circ },120^{\circ }$ and $240^{\circ }$ at three different frequencies. Using multiple modulation frequencies, the unambiguous measurement range can be exceeded [16].

### 2.2. Pulse-Based ToF (PB-ToF)

There are different approaches to realize a pulse based ToF camera [5,17,18]. In principle, a PB-ToF camera emits only a short light pulse, which is reflected in the scene and again captured by a sensor array. The sensor is equipped with one or more optical or electronic shutters, which detect the incident light pulse in one or two very short temporal windows, the *gates*. In case the observed object’s distance relates to the time-of-flight measurable in a given gate, the amount of incident active light is proportional to this object distance. There are two main approaches to implement a PB-ToF camera, i.e., using a shutter setup that realizes a single optical gate or two optical gates [18].

**Figure 2:**
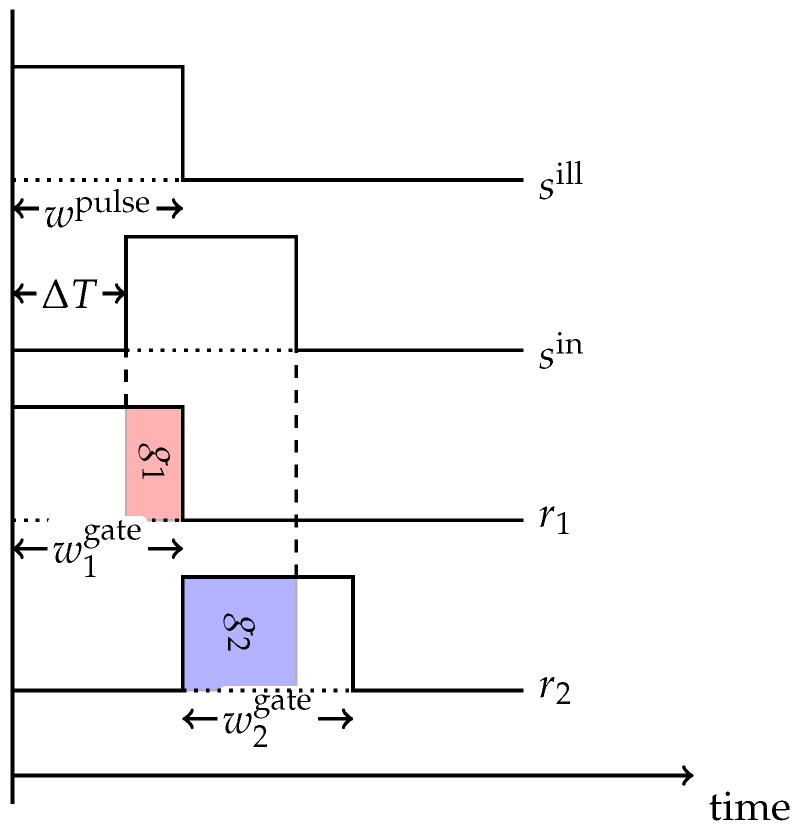
Pulse based camera timings.

In the following, we describe the two-gate approach as realized by the S11963-01CR sensor chip from Hamamatsu Photonics, which is our reference device. This device realizes a *two-gate* PB-ToF approach, very similar to the one described by Davis and Gonzalez-Banos [18]. This kind of sensor accumulates the reflected light in the sensor using two gates, see Figure 2. The first gate $g_{1}$ is activated synchronously with the emitted light pulse. Then, the first gate is closed and synchronously the second gate $g_{2}$ is opened. Due to the distance to an object, the reflected light pulse is shifted by ΔT and the reflected photons will be distributed according to $g_{1}$ and $g_{2}$.

**Figure 3:**
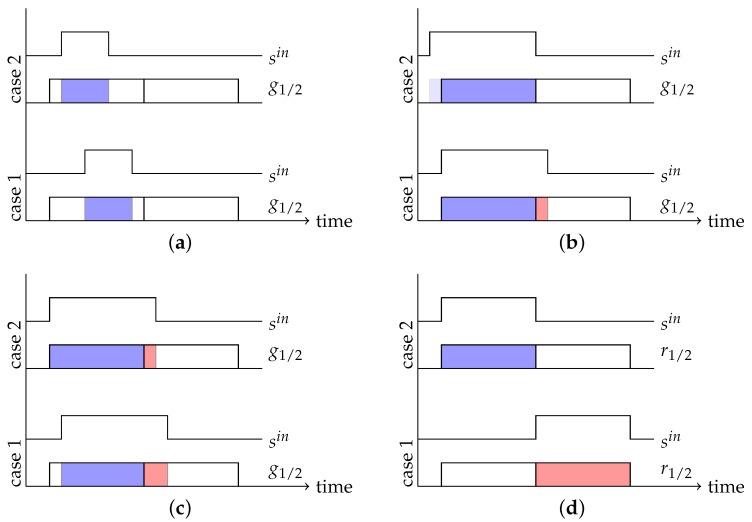
Pulse Based Camera Parameter Choices. (**a**) When $w_{1,2}^{\mathrm{gate}}>w^{pulse}$ the unambiguous range shrinks, as case 1 and case 2 cannot be distinguished; (**b**) When $w_{1,2}^{\mathrm{gate}}<w^{pulse}$ the $\frac{g_{1}}{g_{1}+g_{2}}$ ratio does not change proportional to the distance. The time shift between case 1 and case 2 is the same as in Figure 3c, but the ratio does not change by the same factor; (**c**) See (**b**); (**d**) For $w_{1,2}^{\mathrm{gate}}=w^{pulse}=w$ the gate width relates to the maximum measurable distances.

Table 1 depicts the abbreviations used in the following derivation of the pulse-based approach, see also Figure 2 and Figure 3. Let $s^{\mathrm{ill}}\left(t\right)$ describe the illumination emitted by the illumination unit. The signal incident to the sensor pixel $s^{\mathrm{in}}\left(t\right)$ is a temporally shifted version of $s^{\mathrm{ill}}\left(t\right)$, where the shift ΔT corresponds to the double distance to the object. Furthermore, the signal is damped by a factor γ>0 depending on the objects reflectivity and its distance to the camera according to the inverse square law. Ambient background light a(t)=a, which can be assumed to be constant over the very short exposition time, results in an additive offset, yielding

(3) $$s^{\mathrm{in}}\left(t\right)=\gamma s^{\mathrm{ill}}\left(t-\Delta T\right)+a=\gamma s^{\mathrm{ill}}\left(t-2\frac{d}{c_{0}}\right)+a.$$

In general, the pulse-based approach assumes rectangular signal shapes (see Figure 2). Thus, from Equation (3) we get

(4) $$s^{\mathrm{ill}}\left(t\right)=\Pi_{0,w^{\mathrm{pulse}},I^{\mathrm{ill}}}\left(t\right),\;s^{\mathrm{in}}\left(t\right)=\Pi_{\Delta T,w^{\mathrm{pulse}},\gamma I^{\mathrm{ill}}}\left(t\right)+a$$

where $I^{\mathrm{ill}}$ is the peak intensity of the illumination unit and $w^{\mathrm{pulse}}$ is the pulse width. Π denotes the standard rect function, i.e., $\Pi_{l,w,a}\left(t\right)=a\Pi \left(\frac{1}{w}\left[t-l\right]-\frac{1}{2}\right)$ its scaled-shifted variant with left shoulder at *l*, width *w* and amplitude *a*.

In a similar fashion, the *gate response curves* $r_{i}\left(t\right),\;i=1,2$ for the two gates are assumed to be rectangular with amplitudes $R_{1},R_{2}$, i.e., $r_{1}\left(t\right)=\Pi_{0,w_{1}^{\mathrm{gate}},R_{1}}$ and $r_{2}\left(t\right)=\Pi_{w_{1}^{\mathrm{gate}},w_{2}^{\mathrm{gate}},R_{2}}$, which leads to integrated gate values (with active illumination)

(5) $$\begin{array}{cc} g_{1}^{\mathrm{light}} & =\int_{-\infty }^{+\infty }s^{\mathrm{in}}\left(t\right)r_{1}\left(t\right)\;dt=R_{1}\int_{0}^{w_{1}^{\mathrm{gate}}}s^{\mathrm{in}}\left(t\right)\;dt\\ & =R_{1}\left(\gamma I^{\mathrm{ill}}\min\left\{w_{1}^{\mathrm{gate}}-\Delta T,w^{\mathrm{pulse}}\right\}+aw_{1}^{\mathrm{gate}}\right), \end{array}$$

(6) $$\begin{array}{cc} g_{2}^{\mathrm{light}} & =\int_{-\infty }^{+\infty }s^{\mathrm{in}}\left(t\right)r_{2}\left(t\right)\;dt=R_{2}\int_{w_{1}^{\mathrm{gate}}}^{w_{1}^{\mathrm{gate}}+w_{2}^{\mathrm{gate}}}s^{\mathrm{in}}\left(t\right)\;dt\\ & =R_{2}\left(\gamma I^{\mathrm{ill}}\left(\min\{\Delta T+w^{\mathrm{pulse}},w_{1}^{\mathrm{gate}}+w_{2}^{\mathrm{gate}}\}\right.\right.\\ & \left.\left.-\min\{\max\left\{\Delta T,w_{1}^{\mathrm{gate}}\right\},w_{1}^{\mathrm{gate}}+w_{2}^{\mathrm{gate}}\}\right)+aw_{2}^{\mathrm{gate}}\right). \end{array}$$

The choice of the right parameters for the light pulse width $w^{\mathrm{pulse}}$ and the gate widths $w_{1,2}^{\mathrm{gate}}$ depends on the distance range to be covered by the camera. Choosing $w_{1,2}^{\mathrm{gate}}>w^{\mathrm{pulse}}$ results in a situation in which the depth cannot be unambiguously recovered over the whole possible range (see Figure 3a). Choosing $w_{1,2}^{\mathrm{gate}}<w^{\mathrm{pulse}}$ does not yield ambiguous cases, but unnecessarily complicates distance calculations (see Figure 3b,c). Consequently, pulse width and gate width are set to the same value *w* (see Figure 3d).

Given the range $d_{max}$ as the maximum distance to be covered by the camera, the gate and pulse widths can be computed using Equation (1) as:

(7) $$w=w_{1}^{\mathrm{gate}}=w_{2}^{\mathrm{gate}}=w^{\mathrm{pulse}}=2\frac{d_{max}}{c_{0}}$$

Plugging the gate values from Equation (7) into the gate charge values in Equations (5) and (6), assuming common gate response values $R_{1}=R_{2}=R$ and restricting ourselves to the unambiguous range ΔT≤w we get:

(8) $$\begin{array}{cc} g_{1}^{\mathrm{light}} & =R\left(\gamma I^{\mathrm{ill}}\left(w-\Delta T\right)+aw\right),\\ g_{2}^{\mathrm{light}} & =R\left(\gamma I^{\mathrm{ill}}\Delta T+aw\right). \end{array}$$

As the impact of the ambient light in Equation (8) cannot be eliminated, both gate values are acquired twice, once with and once without active illumination, yielding gate values $g_{i}^{\mathrm{light}}$ and $g_{i}^{\mathrm{dark}}=Raw,\;i=1,2$, respectively. Thus, the final gate values are then given as

(9) $$\begin{array}{cc} g_{1} & =g_{1}^{\mathrm{light}}-g_{1}^{\mathrm{dark}}=R\gamma I^{\mathrm{ill}}\left(w-\Delta T\right),\\ g_{2} & =g_{2}^{\mathrm{light}}-g_{2}^{\mathrm{dark}}=R\gamma I^{\mathrm{ill}}\Delta T. \end{array}$$

Finally, applying $\Delta T=\frac{2d}{c_{0}}$ we solve for ΔT and distance using Equation (1):

(10) $$\Delta T=\frac{g_{2}}{g_{1}+g_{2}},\;d=\frac{1}{2}c_{0}w\frac{g_{2}}{g_{1}+g_{2}}.$$

In practice, achieving perfect rectangular signals is impossible due to hardware limitations. Therefore, we developed a simulator that can substitute any arbitrary function for $s^{\mathrm{ill}}\left(t\right)$ and $R_{1,2}\left(t\right)$ at Section 4.2. Furthermore, a series of several thousands of pulses are accumulated for an individual range image in order to get a sufficient SNR while being still eye-save, see Table 2. Thus, in dynamic scenes the assumption of constant time shifts ΔT and reflectivity γ for each of the accumulated sub-frames can be violated.

### 2.3. Hamamatsu Pulse Modulated ToF Sensor, Standard Operation

**Figure 4:**
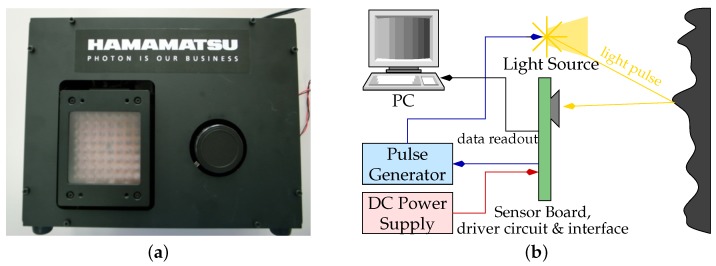
The Hamamatsu PB-ToF prototype. (**a**) External view on the Hamamatsu camera; (**b**) The main components of the Hamamatsu prototype.

In this paper, we perform a detailed investigation of a pulse-based ToF camera prototype which contains a Hamamatsu S11963-01CR sensor chip [20] from Hamamatsu Photonics (Hamamatsu, Japan). Hamamatsu Photonics offers three different PB-ToF chips, two of which are area sensors, and one that is a line sensor, the S11961-01CR, with a total of 256 effective pixels, see Figure 4a. The area sensors have resolutions of 64 × 64 (S11962-01CR) and 160 × 120 (S11963-01CR). For more information on the S11963-01CR; see [21]. Another distributor of PB-ToF cameras is Tridicam, offering a 128×96 pixel area sensor [22] that has been developed at the Fraunhofer IMS and uses Lateral Drift-Field Photodetectors.

### 2.4. Error Sources for ToF Cameras

## 3. Evaluating Generic ToF Error Sources

While error sources for AMCW-ToF cameras have been investigated intensively [4,6,12], PB-ToF range cameras have not been investigated with the same intensity. In this section, we evaluate a PB-ToF prototype with respect to generic ToF error sources.

Our evaluation is based on the prototype provided by the manufacturer Hamamatsu, consisting of the light source, the S11963-01CR imager, driver and driver circuit boards. Further technical details on the imaging electronics of the Hamamatsu device are given in Kawahito et al. [23]. Thanks to the modularity of the prototype, it is possible to modify the light signal pulse in both power and duration. Figure 4b shows the block diagram of the Hamamatsu PB-ToF camera and on the right the casing and the position of light source and the imager [19].

In the context of generic ToF evaluation, we refer to the error sources as discussed by Sarbolandi et al. [4] and build upon the test scenarios presented therein. We restrict ourselves to a subset of tests targeting the most relevant errors appearing in ToF cameras in general. Typical optical effects like shifted optical centers and lateral distortion commonly are estimated using standard intrinsic camera calibration techniques [24]. Beyond these effects, we address the following error sources in this paper, see Sarbolandi et al. [4] for a detailed presentation with respect to AMCW-ToF cameras:

**Temperature Drift:** A drift of the system output, i.e., the distance values in the case of range sensing cameras, is a common effect of many technical devices during the device warm-up. This is due to different effects, i.e., the LEDs and charging gates have different behavior patterns at different temperatures.

Even though this effect mainly reflects the quality of the camera prototype in terms of temperature control, we add this test in order to study the behavior of our system without an active cooling system as a reference.

**Systematic Distance Error:** AMCW-cameras suffer from a systematic error in their depth measurement. For the PB-TOF system, the distance calculation is based on the assumption of correlating a perfect rectangular optical signal $g^{\mathrm{ill}}$ with a perfect rectangular correlation signal $g^{\mathrm{corr}}$. In reality, both signals are not perfect, leading to a systematic error in the depth measurement.

**Depth Inhomogeneity:** At object boundaries, a pixel may observe inhomogeneous depth values. For AMCW-ToF cameras, the processing of superimposed signals caused by light reflected from different depths, yields so-called *mixed pixels* or *flying pixels*. This superposition of different signals leads to incorrect distance values.

Note that flying pixels are directly related to a more general problem, i.e., the multi-path problem; see below.

**Multi-Path Effects:** Multi-path effects relate to an error source common to active measurement systems where the active light is assumed to travel only the direct path from the illumination unit via the object’s surface to the detector. In real applications, additional *indirect light paths* appear, e.g., from light scattering or reflecting in the scene, within the lens systems, or the housing of the camera itself. These multiple responses of the active illumination lead to superimposed signals in a pixel leading to an altered signal and, finally, to wrong distance measurements.

**Intensity-Related Distance Error:** Considering two objects with the same distance to the camera, but with different reflectivity in the relevant NIR range, a reduced SNR is expected for the low reflective object. Beyond this, it has frequently been reported that AMCW-ToF cameras have a non-zero biased distance offset for objects with low NIR reflectivity [25].

**Dynamic Scenery:** One key assumption for any camera-based imaging system is that each pixel observes a single object point during the full acquisition process. This assumption is violated in the case of moving objects or moving cameras, resulting in motion artefacts. AMCW-ToF cameras as well as PB-ToF cameras take several acquisition steps in order to deliver a single range image, i.e., the AMCW-ToF requires several correlation images, while PB-ToF takes two gate images (with a very short temporal gate width) and acquires several thousand pulses in order to collect a sufficient amount of incident light intensity. Thus, in the case of a dynamic scenery, the resulting gate values might be a mixture from different objects or object reflectivity. Processing the acquired gate values while ignoring the motion present during acquisition leads to erroneous distance values at object boundaries.

There are further error sources like the influence of *ambient background light* and *multi-device interference* not investigated in this paper.

### 3.1. Test Scenarios

The error sources described in Section 2.4 are evaluated using the following test scenarios; see also Sarbolandi et al. [4]. These scenarios are related to the discussed error sources according to Table 3.

**Device Warm-Up:** Acquisition of the temporal variation of the delivered range values under constant environmental climate conditions evaluates the *temperature drift*.

**Rail Depth Tracking:** Measurements of the range values delivered by the PB-ToF camera observing a planar wall from various distances are used in order to evaluate the *systematic error* and the *planarity error*, i.e., the out-of-plane error when capturing a planar scene. Additionally, a planar object with varying reflectivity is acquired to evaluate the *intensity related error*.

**Reflective Board:** This setup acquires a reflective board under varying angles and indirect illumination conditions in order to evaluate the influence of *multipath effects*.

**Turning Siemens Star:** Observing a rotating planar object with holes generates various conditions of *motion artefacts* and *flying pixels*.

In the evaluation, we partially compare the Hamamatsu PB-ToF camera prototype with the AMCW Kinect${}^{\mathrm{ToF}}$ camera. Apparently, this is not a fair quantitative comparison, still there is some insight into the qualitative differences between the different ToF principles; see discussion at the end of Section 3.3.

### 3.2. Camera Parameters

It is a challenging task to obtain the camera parameters from a low resolution sensor. Similar to Lindner and Kolb [25] we apply the standard approach by Zhang et al. [24] based on analyzing checker board images. However, we increase the size of the checker board and use a larger number of checkers in order to obtain more robust results. For the given prototype, we yield the parameters stated in Table 4.

### 3.3. Warm up Time Evaluation

This test evaluates the changes in distance measurement due to the warm-up time of the camera. Therefore, the camera is accommodated in a room equipped with an active air conditioner to keep the temperature around 21 ± 0.1 ${}^{\circ }$C. The camera observes a planar wall from an approximately orthogonal viewing direction. Initially we keep the unplugged camera in the room for two hours to make sure it has the same temperature as the room. We start acquiring constantly for two hours. At the beginning of every minute, K=150 frames are saved on the hard disk. The rest of the acquired frames are dropped, but the camera keeps acquiring throughout the whole period.

We define the first set of K=150 frames as reference and compute the average of all frames yielding the *mean frame* $D^{\mathrm{mean}}$. Furthermore, we apply a RANSAC fit to $D^{\mathrm{mean}}$, resulting in a *reference depth frame* $D^{\mathrm{ref}}$. Nevertheless, since the RANSAC is applied to the whole depth frame there is a per-pixel bias with respect to the mean frame. From the mean and the reference depth frame we can calculate the *root mean square error (RMSE)* as

(11) $$\mathrm{RMSE}=\sqrt{\frac{1}{mn}\sum_{x=1}^{m}\sum_{y=1}^{n}{\left(D^{\mathrm{mean}}\left(x,y\right)-D^{\mathrm{ref}}\left(x,y\right)\right)}^{2}}.$$

Moreover, the per-pixel *standard deviation average* (SD) for each sequence of frames $D_{i},\;i=1,\ldots ,150$ is calculated as follows:

(12) $$\mathrm{SD}=\frac{1}{mn}\sum_{x=1}^{m}\sum_{y=1}^{n}\sqrt{\frac{1}{K}\sum_{i=1}^{K}{\left(D_{i}\left(x,y\right)-D^{\mathrm{mean}}\left(x,y\right)\right)}^{2}},$$

where (x,y) and n,m denote pixel coordinates and the camera resolution in *x*- and *y*- direction, respectively.

**Figure 5:**
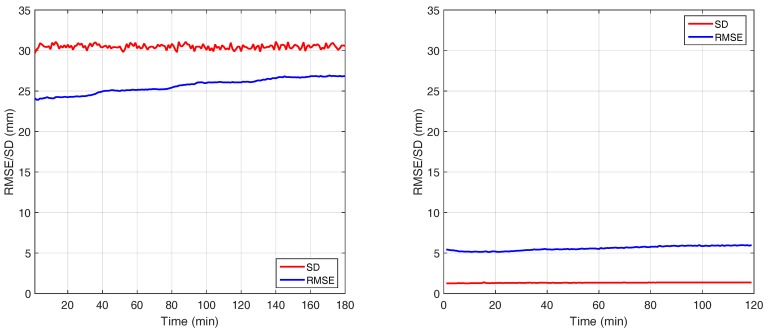
Temperature drift: The Hamamatsu PB-ToF (left) and the Kinect${}^{\mathrm{ToF}}$ camera (right).

Figure 5 shows the result of the device warm-up evaluation of the Hamamatsu PB-ToF camera in comparison to the AMCW Kinect${}^{\mathrm{ToF}}$ camera. As expected, the PB-ToF prototype cannot catch up with the high quality of the commercial Kinect${}^{\mathrm{ToF}}$ device in terms of absolute RMSE and SD as well as in terms of temporal stability. On the qualitative level, we find that the Hamamatsu PB-ToF prototype camera has a significantly higher level of noise (SD). However, the Hamamatsu camera illumination is far less powerful. We quantified the optical power of both systems, the Kinect${}^{\mathrm{ToF}}$ camera and the Hamamatsu PB camera. We measure the direct radiant emittance of both devices at a distance of 80 cm with a Newport 818-SL power meter. The resulting mean optical power of the Kinect${}^{\mathrm{ToF}}$ camera is 283μW/cm${}^{2}$, whereas the Hamamatsu camera emits only 3.9 μW/cm${}^{2}$. However, this is still only a qualitative indication and due to the very different modes of operation and hardware realization it is not possible to convert any quantified values from one device to the other.

### 3.4. Linear Rail

The setup comprises the Hamamatsu camera mounted on a motorized linear rail which slides perpendicular to a white wall at a measurement distance between 0.7 m and 4.2 m and a step-size of 2 cm. As the wall does not cover the full range image for farther distances, we restrict our evaluation to a region-of-interest including pixels lying on the white flat wall in the full distance range. For evaluation, we observe three pixels along a line-of-interest from the image center to the top-left corner which are always covering the wall. The picked pixel positions are (3, 4) (corner), (30, 40) (middle) and (60, 80) (center). We acquire 150 frames for each distance. In order to re-project the range values into 3D-space, we used the parameters depicted in Table 4.

**Figure 6:**
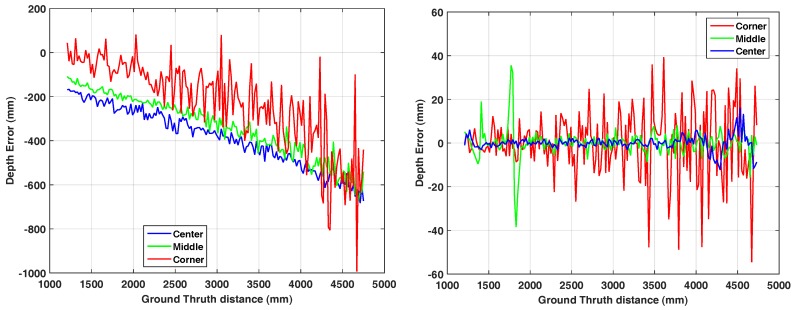
Linearity error, raw data (left) and per pixel corrected (right).

Figure 6 (left) shows the results of this linearity error of the three pixels. Similar to Theiß [26], the error of all three points keeps growing according to the distance to the wall.

#### Per Pixel Correction

Apparently, the linearity error is not random but it shows a trend both over distance and pixel position on the image. Therefore, we apply a per pixel error correction over distance. We utilized *cubic spline interpolation* applied to 100 range measurements for each pixel to estimate the distance error. The error is then compensated on a new measurement and shown on Figure 6 (right). As expected, the method removes the offset error but the noise-related deviation remains uncorrected.

### 3.5. Planarity

In each range image acquired on the rail, there is a region that lies on the flat white wall, so the resulting range values should ideally result in a plane. Similar to Khoshelham and Elberink [27], we apply a RANSAC plane fitting algorithm to ignore outliers and calculate the standard deviation of the points from the fitted plane as the planarity error.

**Figure 7:**
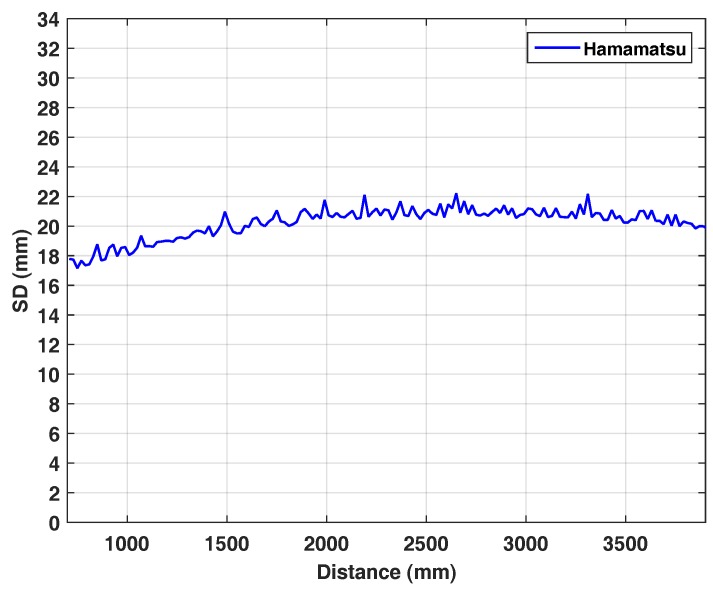
Planarity error. The standard deviation of the 3D points within the range-of-interest for the Hamamatsu.

Figure 7 shows the planarity error as SD over distance. Interestingly, the planarity characteristics of the Hamamatsu camera remains constant in the range of 18–22 mm over 3.5 m distance. At this point, we omit a comparison to Kinect${}^{\mathrm{ToF}}$, as the higher noise level of the Hamamatsu camera (see Figure 5, left) makes the SD-values incomparable. Quantitatively, however, we can observe, that for the Kinect${}^{\mathrm{ToF}}$ the SD-values are increasing over distance (see Figure 16 in [4]).

### 3.6. Intensity Related Error

In theory, the intensity of the reflected active light should affect the standard deviation of the range measurement for AMCW or PB ToF only. As the gate charge amplitudes of the light reflected from the object cancel each other out (see Equations (2) and (9)), the mean value should stay unchanged. However, Lindner and Kolb [25] measured intensity related errors of up to 50 mm for an early 19k AMCW-ToF prototype camera from pmd technologies (see Figure 4 in [25]). As both prototype cameras, the pmd technologies’ 19k and the Hamamatsu prototype camera, have not been optimized for this error, we expect to observe a similar behavior for the PB ToF camera.

Similar to [28] we evaluated the camera using an intensity checker board which is a 2×5 checkerboard with varying gray levels at 1.2 m distance. To cancel the temporal noise, we worked on the average of 50 consecutive frames. The checkerboard has been printed using a standard laser printer which delivers sufficiently proportional reflectivity in the visual and the NIR range.

**Figure 8:**
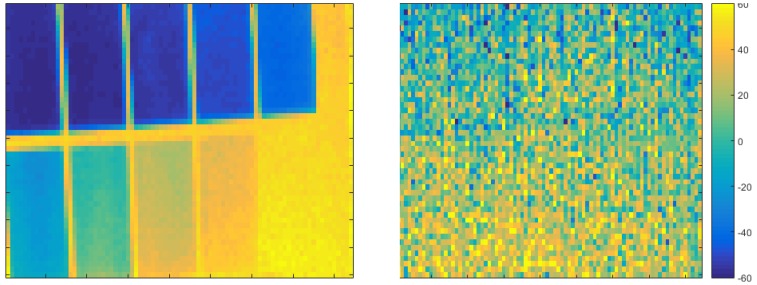
Intensity checker board (left) and depth image from the same view (right). The depth value is given in mm, measured at a distance of 1200 mm.

Figure 8 compares the intensity image using $g_{1}+g_{2}$ with the range image. Again, a direct comparison to the Kinect${}^{\mathrm{ToF}}$ camera is not feasible due to the high noise level. Unlike the intensity related bias observed for the pmd technologies’ 19k, there is no visible systematic intensity-related error for the Hamamatsu PB-ToF camera. For now, we do not have any technological explanation for this different behavior.

### 3.7. Turing Siemens Star

Every imaging system is prone to motion artefacts in case of dynamic scenery. This is mainly due to the acquisition time that is required to gather enough light from the scene, during which the scene should remain still. The Hamamatsu prototype camera acquires 4 pulse cycles of 75 ns each, which are collected 3000 times per depth measurement (see Table 2).

We use the turning Siemens star setting [4] to evaluate the motion artefacts and flying pixels of the Hamamatsu PB-ToF prototype. Here, the camera is set up in front of a Siemens star mounted to a stepper motor to control the rotating speed of the star. The background is a homogeneous white wall. For several speeds ranging from 0,…,120 revolutions per minute (RPM), the camera records 200 depth frames.

**Figure 9:**
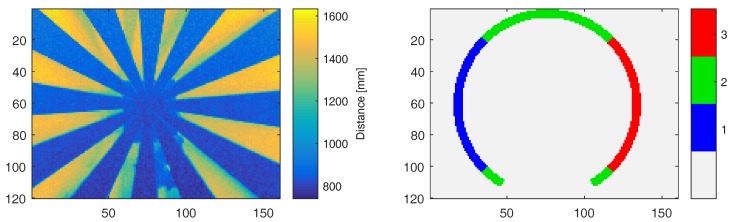
Turning Siemens star depth data example (left) and segments used in the evaluation (right).

Circular segments as regions of interest are manually selected in the images so that the area of foreground and background are equal from the camera perspective. Based on the camera orientation, three different areas are defined that correspond to horizontal (2) and vertical movement (1, 3); see Figure 9, right. Thus, we can distinguish different relative configurations between light source and imager. In order to identify ground truth foreground and background distances, we acquire the depth image for the static scene (steady turning star), manually segment foreground and background regions, and apply plane fitting to these regions in 3D. Now, we can use thresholding in order to classify individual pixels as foreground and background. For the turning wheel, we classify each range measurement in the regions of interest as foreground or background if the range values lies within 20% distance to the reference depths. Otherwise, the depth value is considered invalid. Since we observe a circular segment, we convert the RPM values into *pixel per second*, which makes the results easier to compare.

**Figure 10:**
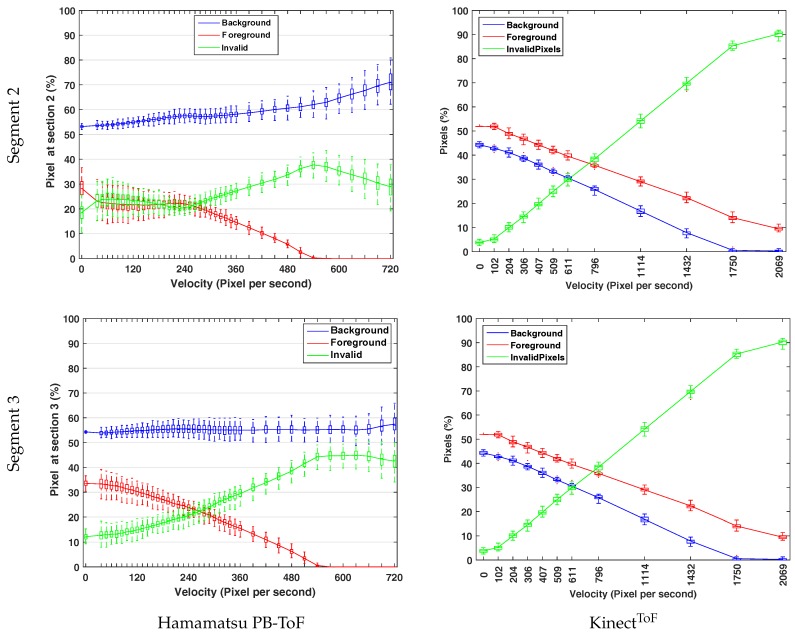
Turning Siemens star: Motion artefacts statistics for the Hamamatsu PB-Tof camera (**left column**) and the Kinect${}^{\mathrm{ToF}}$-camera (**right column**) for section 2 (top row) and 3 (bottom row) (see Figure 9).

Figure 10 shows the statistics of the foreground and background for the vertical region Section 2 (Figure 10, top row) and the horizontal region Section 3 (Figure 10, bottom row). As the illumination unit and the imager are horizontally aligned in the Hamamatsu prototype (Figure 10, left column), we expect more occlusion on vertical slots, i.e., in Section 2. This effect is visible in the results, especially for low RPM values. Comparing the classification results, i.e., the flying pixels, the share of invalid pixels in the horizontal Section 2 starts from about 18%, which is about 1.5 times larger than in the vertical Section 3. As the velocity increases in both sections, the amount of foreground pixels decreases, whereas a significant and moderate increase for invalid and background pixels can be observed, respectively.

Even though a direct comparison between an AMCW Kinect${}^{\mathrm{ToF}}$ camera and the Hamamatsu PB-ToF prototype is not very reliable due to an unknown invalid pixel classification of the Kinect${}^{\mathrm{ToF}}$ (compare Figure 22, right column, in Sarbolandi et al. [4]) (Note, that in Figure 22 in Sarbolandi et al. [4] there is a glitch in the x-labels for the rotation speed for the turning star experiment with the Kinect${}^{\mathrm{ToF}}$. Instead of 91 px/s, the max. speed is 2096 px/s. Note that the 120 RPM velocity for both cameras result in different px/s value due to the different resolution of both cameras.), we present the differences in a qualitative manner. For 0 speed, the initial foreground/background estimates show that the Kinect${}^{\mathrm{ToF}}$ slightly overestimates foreground and underestimates background (both ≈10% relative error), while the Hamamatsu significantly underestimates foreground (≈30% relative error) and slightly overestimates background (≈10% relative error). For higher speed, the Kinect${}^{\mathrm{ToF}}$ always delivers slightly more foreground than background pixel and an increasing amount of invalid pixels. The Hamamatsu PB-ToF prototype delivers a slightly increasing amount of background pixel, a decreasing amount of foreground pixel, and an increasing number of invalid pixels (categorized using the 20% threshold; see above) with increasing speed. In comparison with the Kinect${}^{\mathrm{ToF}}$, the Hamamatsu camera delivers more invalid than foreground pixels at 260 px/s, where this parity appears about 800 px/s for the Kinect${}^{\mathrm{ToF}}$. However, the Hamamatsu camera maintains the percentage of background pixels up to 720 px/s where Kinect${}^{\mathrm{ToF}}$loses 15% already at this speed.

### 3.8. Reflective Board

The distance computation in active imaging systems such as AMCW- or PB-ToF devices is based on the assumption that the light that is captured by a single pixel originates from a single light path from the illumination unit to the object point observed by the pixel which, in turn are assumed to have a homogeneous depth. This assumption is violated at object boundaries, yielding flying pixels, but also when light additionally travels different, indirect paths due to scattering and reflection in the scene or the camera itself.

**Figure 11:**
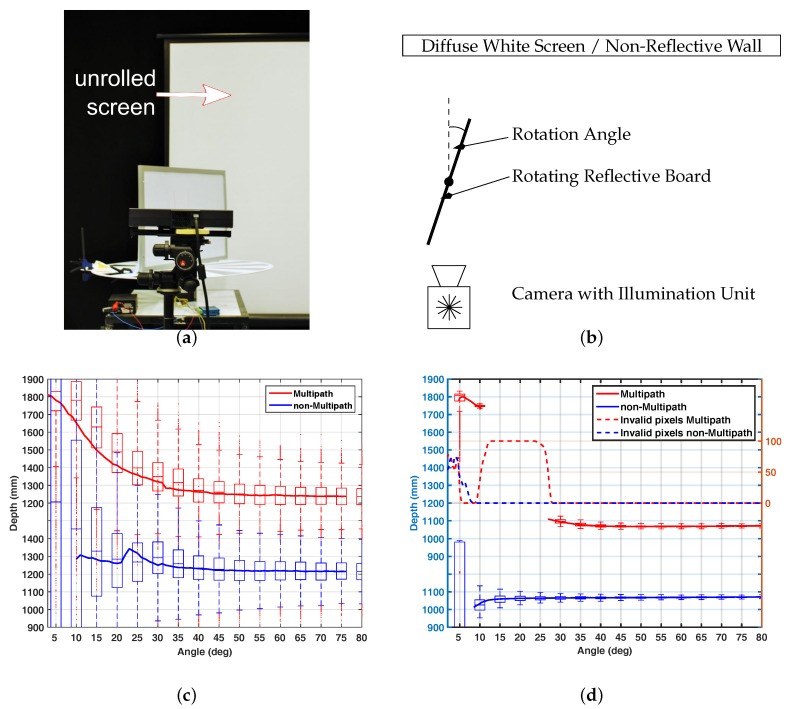
Reflective board scenario setup (photo (**a**) and top-view (**b**)) and the multipath results for the Hamamatsu camera (**c**) and the comparison to the (**d**). (**a**) Reflective Board Setup, Photo; (**b**) Reflective Board Setup, Top View; (**c**) Result for Hamamatsu PB ToF; (**d**) Result for Kinect${}^{\mathrm{ToF}}$.

Figure 11a,b show the setup of the test scenario. As the multipath effect depends not only on the reflectivity of the objects in the scene, but also on the orientation of the objects to each other and to the camera, the main concept of the evaluation setup is to vary the angular orientation of a reflective object with respect to a light scattering background. The range camera observes a reflective whiteboard of 60×40 cm size which is vertically placed on a turning table. The whole setup is located in front of a low reflective wall at some 170 cm distance (*non-multipath* variant). Indirect light is optionally inserted by uncoiling a white projector screen directly in front of the wall (*multipath* variant). The vertical board is rotated from $0^{\circ }$ to $90^{\circ }$ with resolution of $0^{\circ }15^{\prime }$. For each step we acquire 20 frames. For evaluation, only the points lying on the rotation axis are considered, as they remain at the same distance to the camera.

Figure 11c shows the result of the reflective board scenario for the Hamamatsu PB-ToF prototype. Additionally we give the result for the AMCW Kinect${}^{\mathrm{ToF}}$ camera in Figure 11d. Note that due to setup variations, the distance to the rotation axis of the board is not the same for both devices. It can be seen that for the non-multipath scenario, the PB-ToF prototype delivers less stable distance results, i.e., a high SD; see discussion in the device warm-up scenario Section 3.3. Most likely, this explains the variation of the mean distance for low angles for the PB-ToF prototype as well. In the multipath scenario, the Kinect${}^{\mathrm{ToF}}$ camera does not deliver all data, as the device partially detects multipath corrupted pixels, i.e., there are no results between angles of $22^{\circ }$ and $10^{\circ }$. Compared to the Kinect${}^{\mathrm{ToF}}$ camera, the PB-ToF prototype delivers very comparable data in terms of mean values.

## 4. Specific Pulse-Based Effects

Beyond the generic ToF related error sources evaluated in Section 3, this section discusses specific aspects of pulse-based ToF cameras. In Section 4.1, the behavior of the gate values $g_{1,2}$ as a function over distance and intensity is investigated. Based on the results, and in combination with standard range calibration techniques, we discuss options to improve on the standard deviation of the distance values of the PB-ToF cameras. In Section 4.2 we estimate signal shapes for the illumination $s^{\mathrm{ill}}$ and the gate responses function $r_{1,2}$ and simulate their influence on the systematic error and validate our model against real-world measurements with our Hamamatsu PB-ToF camera.

### 4.1. Optimized Operation Range for Calibrated PB Range Data

In this section we give a brief overview on the gate values as a function of distance and intensity with the ultimate goal of reducing the standard deviation of the final distance values delivered by the PB ToF camera after depth calibration. The main approach for operation range optimization consists of three steps:

1. measure gate values and their standard deviation separately as a function of distance and of intensity,
2. apply the required non-linear calibration to the range data and the the distance- and intensity-related gate values, and
3. shift the operation range to a region with less standard daviation in gate values.

Note that this approach is, in general, also applicable to AMCW ToF cameras.

#### 4.1.1. Gate Values as a Function of Distance and Intensity

**Figure 12:**
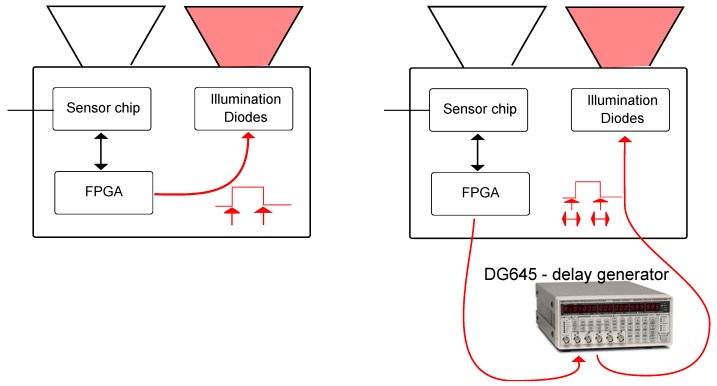
Modifying the prototype camera using an external Delay generator.

**Figure 13:**
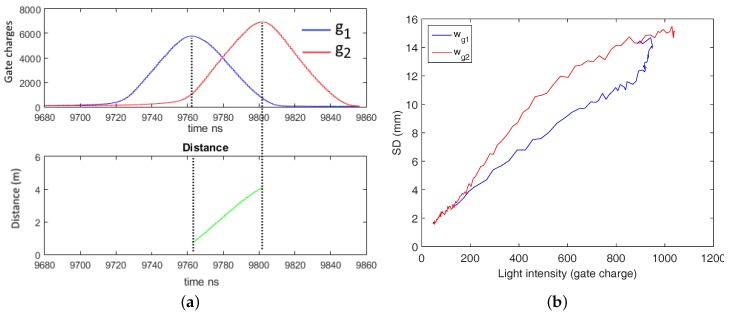
Gate values as a function over distance at constant light intensity (**a**) and the SD for the gate values (**b**). (**a**) Gate charges and distance values; (**b**) SD of gate charges over intensity.

In a first step we acquire the gate values $g_{1,2}$ *independently* as a functions of distance and intensity. Therefore, we modify the Hamamatsu PB-ToF prototype’s illumination control by inserting an external delay generator; see Figure 12. Adding an additional delay, we shift the light pulse over the gates in the time domain, i.e., we simulate a distance change proportional to the delay applied yielding a *virtual distance*. We place the camera in front of a planar wall at a distance of 1.2 m and shift the pulse over the full width of both gate windows. For every distance-intensity value, we acquire 50 frames and average a 10×10 pixel block in the center of the frame to reduce the noise effect. Figure 13a shows the gate values as functions over distance, as well as the calculated distance (left-bottom). Apparently, the peak intensities of both gate functions differ significantly. We cross-check this effect on other pixel regions and validate it with some local variations.

In a second step, we additionally control the intensity of the illumination unit by varying the voltage applied to the illumination unit. As it is rather difficult to measure the light incident to the sensor, we relate the captured gate values to the intensity observed by the imager, i.e., to $I^{\mathrm{in}}=g_{1}+g_{2}$. As an overall result we obtain each gate value as a 2D function over distance and intensity, i.e., $g_{1,2}\left(d,I^{\mathrm{in}}\right)$. Unfortunately, the intensity range of this setup is limited due to the restrictions in the dynamic voltage range applicable to the LEDs. Due to the exponential increase of forward current of P-N junction diodes [29] it is a challenging task to linearly dim the LEDs. Hence, gate values and intensities are always measured in arbitrary digital units, as delivered by the Hamamatsu PB-ToF camera.

**Figure 14:**
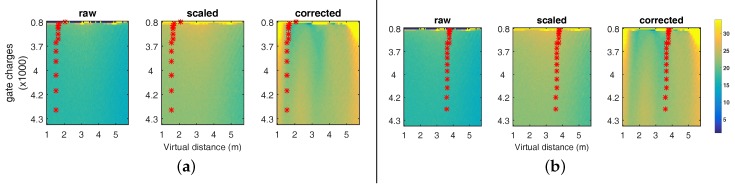
2D SD diagrams for the raw, the linearly mapped and the calibrated data (left to right). The red dots indicate the location of some of the rail measurements in the distance-intensity plane for the initial operation range (**a**)) and the shifted operation range (**b**). (**a**) 2D SD diagrams for standard operation range; (**b**) 2D SD diagrams for shifted operation range.

Figure 13b shows the standard deviation of the gate charges as a function over intensity. The intensity-distance related standard deviation are shown in Figure 14 and are discussed in the next section. Apparently, the SD of the intensity is not fully proportional to the square root of light intensity as it would be expected in case of dominant shot noise. We assume that this is due to the prototype nature of the camera system at hand, which might be more influenced by electronic and quantization noise.

#### 4.1.2. Optimizing the Operation Range

In this section, we investigate the SD on calibrated range data and the option to virtually shift the operation range of the camera in order to improve its SD behavior in a distance range of interest. As the raw range data is distorted by systematic range error (see Section 2.4), any optimization by shifting the operation range needs to take the transformation due to range error calibration into account. For calibration of the range data, we use a simple two-stage process. In the first step, we linearly map the measured distance range to the real (reference distances) and, in a second step, we apply per-pixel curve fitting to the remaining error, yielding the calibrated data (see Section 3.4).

As we need consistent data for the real-world evaluation of the SD for uncalibrated and calibrated data and the gate values acquired in Section 4.1.1, we set up another rail measurement, this time on a short range of only one meter. The rail shift starts from 1 m to 2 m distance to the wall and in every second centimeter, 50 frames are acquired and averaged. The initial average range error and SD for the uncalibrated raw data at 2 m is 600 mm and 80 mm, respectively. For the calibrated data these values are reduced to 2 mm and 40 mm, respectively.

Figure 14 ‘raw’ shows the intensity-distance SD diagram for the raw data including distance-intensity samples belonging to the one meter real measurement. As the linear mapping and the calibration alter the range values, the SD values are altered as well (see Figure 14 ‘scaled’ and ‘corrected’).

**Figure 15:**
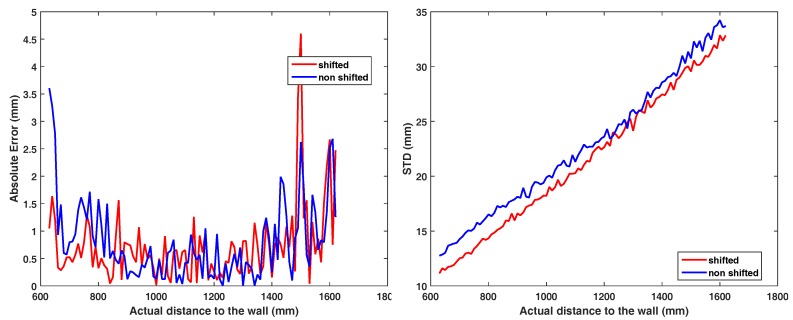
Distance error for the non-shifted and the shifted range measurement (left) and the corresponding SD improvement (right).

Due to the non-linear calibration transformation, the region between 3 m and 4 m exhibits less SD than other distance regions (Figure 14a ‘corrected’). Using the external pulse delay (see Section 4.1.1), we can virtually move the operation range of the camera. In our case, we add a 6.6 ns delay to the light pulse which corresponds to a 2 m distance offset, shifting the measurement of the rail in the range of [3,4] m. Figure 14b shows the resulting operation range after redoing the 1 m rail measurement. This shift influences the absolute RMSE of the calibrated range only marginally, i.e., we have an RMSE of 1.079 mm before and of 1.001 mm, which is a small improvement; see also Figure 15, left. Figure 15, right, gives the effect on the SD in range values. We observe a moderate improvement of 1.3 mm in average between 600 and 1600 mm and maximum of 2.2 mm at 820 mm.

### 4.2. Signal Shapes and Simulation

**Figure 16:**
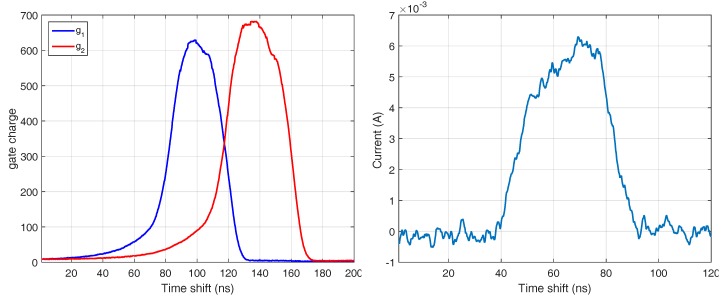
Sampled gate response using 4ns light pulse (left) and measured light pulse shape using fast diode (right).

As we mentioned in Section 2.2, the emitted light pulse $s^{\mathrm{ill}}\left(t\right)$ and the shutter response $r_{1,2}\left(t\right)$ are distorted with respect to the trigger signal by the electronic components. Figure 16, left, shows the light pulse shape measured by a fast diode according to the shutter timings. Note that the shutter signals do not represent the responses of the shutter but only the trigger timings. However, determining the response curve of the gates is not as straightforward as the acquisition of the light pulse using a fast photo diode. Therefore, we programmed the camera so that it emits 4 ns light pulse which was the shortest possible pulse using the LEDs and shift it with the resolution of 200 ps until both shutters were fully closed (Figure 16, right).

**Figure 17:**
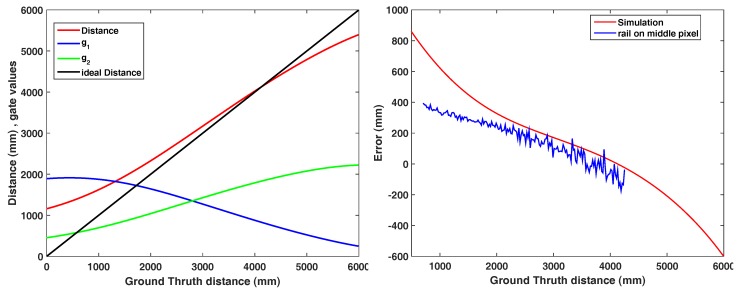
Simulated distance (left) comparison of distance error on simulated and rail measurement (right).

A PB camera simulator based on the mathematical model of Section 2.2 is designed to simulate the output of the camera based on the imperfect light signal of i(t) and the shutter response $r_{1,2}\left(t\right)$. The pulse inputs can be the outcome of a measuring device (Figure 17, left). According to the simulator, the simulated error is computed based on the ideal line. The simulation shows a distance error symmetric to the center distance (3 m) due to the pulse distortions mainly on the edges and Figure 17, right, compares the error from the simulation approach and the rail measurement on the middle point from Figure 6.

## 5. Conclusions

In this paper, we present an in-depth evaluation of a pulse-based (PB) ToF camera prototype based on the Hamamatsu area sensor S11963-01CR. We evaluate the prototype with respect to temperature drift, systematic depth error, depth inhomogeneity, multi-path effects, and motion artefacts. Even though the noise level of the prototype is very high compared to commercial products like the Kinect${}^{\mathrm{ToF}}$ (see Figure 5), the quantitative comparison indicates promising results and, thus, significant development potential for PB-ToF cameras:

- Distance error in the image center is, after simple per pixel distance correction, in the range fairly below 1 cm up to 4 m distance (Figure 6, right).
- Planarity error is around 2 cm and, due to the measurement principle (see Section 4.1), fairly constant over distance (Figure 7).
- The intensity-related error is, even for this PB-ToF prototype, less prominent compared to early AMCW devices (compare [25]).
- There are apparent motion artefacts with a significant loss in foreground pixel at jumping edges for higher speed. Background pixels, however, are detected with significant higher robustness.
- The multi-path interference of the PB-ToF prototype is comparable to the current commercial Kinect${}^{\mathrm{ToF}}$ device in terms of mean range error.

Beyond the evaluation of the PB-ToF prototype, we further investigated the prototype with respect to the observed range measurement errors. Therefore we measured the gate values, and thus (virtually) measured distance deviations as a function of distance and intensity. Furthermore, we used the gate response and the illumination signal in order to simulate the PB-ToF prototype. The results can be summarized as follows:

- The response curves of both gates are not perfectly symmetric (Figure 13a). Combined with the gate response and the illumination signal (Figure 16), our simulation reproduces the measured depth deviation with good quality (Figure 17).
- Using distance deviations as a function of distance and intensity, we evaluated the effect of the calibration of the range values (correcting for the systematic error) on the resulting standard deviation. As the non-linearity of the calibration alters the standard deviation (Figure 14), we suggest to tune the range measurement in order to reduce the standard deviation (Figure 15). This approach requires pre-knowledge about the range and intensity ranges expected to be measured in a specific scenario.

## Figures and Tables

**Table 1:** Symbols used for pulse-based ToF.

$s^{\mathrm{ill}}$	emitted illumination signal
$s^{\mathrm{in}}$	illumination signal incident to sensor
$r_{1},r_{2}$	gate response function
$g_{1}^{\mathrm{dark},\mathrm{light}},g_{2}^{\mathrm{dark},\mathrm{light}}$	gate values w. and w/o active illumination
$w^{\mathrm{pulse}}$	pulse width
$w_{1}^{\mathrm{gate}},w_{2}^{\mathrm{gate}}$	gate width for gate 1, 2
ΔT	time shift between $s^{\mathrm{ill}}$ and $s^{\mathrm{in}}$
γ	damping due to distance & scene reflectivity

**Table 2:** Camera parameters, see also [19].

Parameter	Unit	Default Value	Description
light pulse width $w^{\mathrm{pulse}}$	ns	30	Duration of the light pulse
light pulse delay	ns	75	Delay between light pulse emission and opening of the first shutter to incorporate the hardware delay of the illumination or to shift the measurement range
$w_{1,2}^{\mathrm{gate}}$	ns	30	Opening time of the two shutters
$w^{\mathrm{close}}$	ns	9940	Time at which both shutters are closed between pulses
tacc	ns	10,000	Total acquisition time for a single frame which is equal to $w^{\mathrm{pulse}}+w^{\mathrm{gate}}+w^{\mathrm{close}}$
numPulse	–	3000	Number of pulses per range measurement
FPS	1/s	≈5–10	Frames per second

**Table 3:** The relation between the different ToF effects to the designed test scenarios. Each test addresses primarily one or two separable effects denoted by •.

Test-Scenarios∖Effect	Temp. Drift	System. Error	Depth. Inhomog.	Multipath Effect	Intens.-Rel. Error	Dynamic Scenery
Device Warm-Up	•					
Rail Depth Tracking		•			•	
Reflective Board				•		•
Turning Siemens Star			•			

**Table 4:** Intrinsic parameters of the Hamamatsu pulse-based ToF prototype. The distortion is given using radial parameters $\left(k_{1};k_{2};k_{3}\right)$ and tangential parameters $\left(p_{1};p_{2}\right)$ according to Zhang’s model [24].

Resolution (x,y):	160×120 px
Focal length (x,y):	(259.69,259.62) mm
Principal Point (x,y):	(81.01,54.97) px
Distortion:	(−0.735 , 0.826 , −1.046 , 0.014 , −0.001)
	$\left(k_{1},k_{2},k_{3},p_{1},p_{2}\right)$
